# Association of phenotypic age and accelerated aging with severity and disability in patients with acute ischemic stroke

**DOI:** 10.1016/j.jnha.2024.100405

**Published:** 2024-11-02

**Authors:** Yongkang Liu, Jiangchuan Wang, Zicheng Wei, Yu Wang, Minghua Wu, Jianhua Wang, Xiao Chen, Rong Chen

**Affiliations:** a Department of Radiology, Affiliated Hospital of Nanjing University of Chinese Medicine, Nanjing 210029, China; b Encephalopathy Center, Affiliated Hospital of Nanjing University of Chinese Medicine, Nanjing 210029, China; c Department of Diagnostic Radiology and Nuclear Medicine, University of Maryland School of Medicine, 100 N Greene, Baltimore, MD 21201, United States

**Keywords:** Acute ischemic stroke, Phenotypic age, Severity, NIHSS, Disability

## Abstract

**Objective:**

Biological age may be more accurate than chronological age in determining chronic health outcomes. However, few studies have shown the association between biological age and acute ischemic stroke (AIS). In this study we showed the association between phenotypic age (PhenoAge) or accelerated aging and severity and disability in patients with AIS.

**Design:**

Retrospective study.

**Setting and subjects:**

936 patients with AIS during January 2019 to July 2021 and 512 patients during June 2022 to July 2023 for a validation.

**Methods:**

Stroke severity was evaluated based on the National Institute of Health stroke scale (NIHSS) questionnaire scale. Disability was evaluated by modified Rankin Scale. PhenoAge was calculated based on chronological age and 9 clinical chemistry biomarkers. Logistic regression analyses were applied to estimate the relationship between PhenoAge and the severity and disability.

**Results:**

PhenoAge (odds ratio [OR] = 1.03, 95% confidence interval [CI]: 1.0–1.04, for NIHSS ≥ 5; OR = 1.05, 95%CI: 1.03−1.07, for NIHSS ≥ 10) was independently associated with stroke severity. The probability of NIHSS ≥ 5 or NIHSS ≥ 10 was significantly increased in individuals with accelerated ageing versus individuals with no accelerated aging (age gap: OR = 1.79, 95%CI: 1.18−2.72; OR = 3.53, 95%CI: 1.60−7.77; phenotypically older vs. phenotypically younger: OR = 2.01, 95%CI: 1.21−3.35; OR = 3.69, 95%CI: 1.36−10.0). Similar trends was observed when accelerated aging was defined by residual discrepancies between PhenoAge and chronological age (OR = 1.02, 95%CI: 1.01−1.04, for NIHSS ≥ 5; OR = 1.05, 95%CI: 1.02−1.08, for NIHSS ≥ 10). The area under the curve of PhenoAge was higher than that of chronological age in identifying patients with NIHSS ≥ 5 (0.66, 95%CI:0.62−0.70 vs. 0.61, 95%CI: 0.58−0.65, p < 0.01) and NIHSS ≥ 10 (0.69, 95%CI:0.60−0.77 vs. 0.63, 95%CI: 0.55−0.72, p = 0.05). The probability of severe disability was significantly increased in individuals with accelerated aging versus individuals with no accelerated aging (age gap: OR = 2.87, 95%CI: 1.09−7.53; phenotypically older vs. phenotypically younger: 4.88 (1.20−19.88). Similar results were observed in the validation population.

**Conclusion:**

PhenoAge or accelerated aging is associated with stroke severity and disability even after adjusting for chronological age.

## 1 Introduction

Acute ischemic stroke (AIS) is a major cause of mortality and disability globally [1]. Nearly 800,000 patients suffer from stroke in the United States annually, of whom approximately 700,000 are AIS victims [2]. Since 2015, the burden of stroke in China has been on an alarming growth trend, making it the leading cause of death and disability [3,4]. Acute damage to brain cells may lead to series of signs and symptoms. Approximately 50% of AIS survivors exhibit appendicular sensory or motor dysfunction, cognitive deficits, emotional disorders [5], resulting in adverse implications for quality of life and long-term disability [6]. Therefore, AIS has been considered as a serious and life-threatening disease worldwide.

Stroke severity is associated with the prognosis and clinical outcome [[7], [8], [9]]. The National Institutes of Health Stroke Scale (NIHSS) is a well-established tool for quantifying the severity of ischemic stroke [[10], [11], [12]]. NIHSS scores range from 0 to 42, with a higher total score indicating a more severe neurological deficit [13]. Although the NIHSS was mainly utilized to measure neurological deficit rather than functional outcome, increasing evidence suggests that a high total NIHSS score correlates with poor outcomes in stroke patients [[7], [8], [9]]. Predicting severity is beneficial for assessing outcomes following AIS. Recently, some studies have focused on determining the associated factors of stroke severity [[14], [15], [16], [17], [18]].

Phenotype Age (PhenoAge) is a novel biological aging measure based on a Gompertz mortality model that incorporates chronological age and 9 clinical chemistry biomarkers [19,20]. Phenotype age may be more accurate than chronological age in determining chronic health outcomes [21]. It has been widely employed in various studies in recent years. PhenoAge was robustly associated with mortality [22], Coronavirus disease 2019 (COVID-19) severity [23]. PhenoAge was also significantly associated with systemic inflammation, frailty, and cardiovascular disease [24] and common chronic respiratory diseases and decreased lung function [25]. All these findings above mentioned suggest PhenoAge or accelerated aging may serve as a useful tool for disease assessment in clinical research.

Several studies have shown that biological age, estimated based on DNA methylation, is associated with 3-month mortality in ischemic stroke or stroke recurrence [26,27]. However, whether PhenoAge can partly imply the severity and disability of AIS remains to be clarified. We hypothesized that PhenoAge or accelerated aging may be associated with stroke severity and disability. Therefore, the current study aims to explore the association between PhenoAge and the severity of AIS, aiming to find a promising indicator for the administration and management of AIS. Additionally, we evaluate the association between accelerated aging and the probability of severe disability at discharge.

## 2 Methods

### 2.1 Participants

**Fig. 1:**
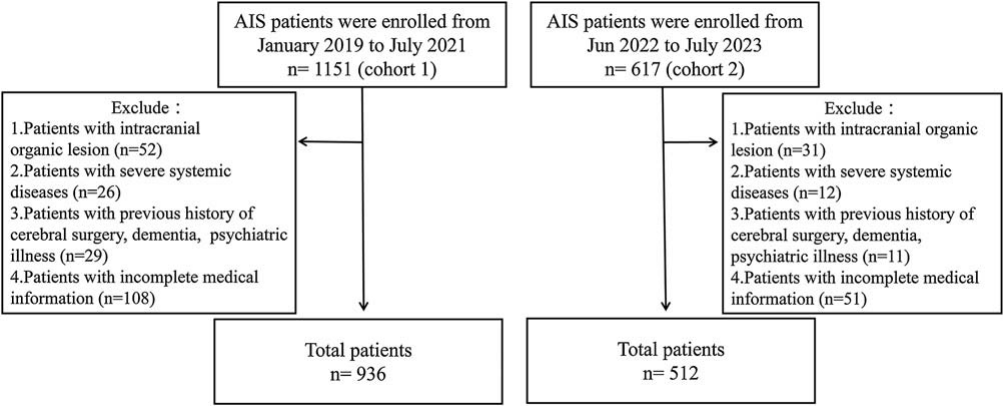
The flowchart of study population. 936 patients in cohort 1 were included to show the association between PhenoAge and stroke severity and disability. 512 patients in cohort 2 were included for a validation.

This retrospective study was approved by the Ethics Committee of the Affiliated Hospital of Nanjing University of Chinese Medicine. Informed consent was waived by the Ethics Committee of the Affiliated Hospital of Nanjing University of Chinese Medicine because of the retrospective design. The patients with acute ischemic stroke in the Encephalopathy Center of our institution during January 2019 to July 2021 were included. All the participants presented with new focal neurological dysfunction within 24 h of symptom onset were diagnosed as AIS. The inclusion criteria were as follows: (1) age ≥18 years; (2) acute lesions detected on diffusion weighted imaging (DWI). The exclusion criteria were as follows: (1) intracranial organic lesion, such as hemorrhage or tumor; (2) severe liver, kidney, respiratory or circulatory system dysfunctions; (3) previous history of cerebral surgery; (4) previous history of dementia, psychiatric illness; (5) incomplete medical information. The demographics, general clinical data, biochemical results and radiological data were collected from medical records. Finally, 215 patients (mean age, 67 years; women, n = 77) were excluded and a total of 936 patients were enrolled in the current study as a training group. Additionally, another 512 AIS patients from Jun 2022 to July 2023 were enrolled as a validation group. The inclusion criteria and exclusion criteria were same as those in training group. The study flowchart is shown in Fig. 1. All the AIS patients underwent routine medical intervention including antiplatelet, anticoagulants, statin therapy. Some of them with large vessel occlusion underwent thrombolysis or endovascular therapy.

### 2.2 Collection of general data

The general baseline characteristics involving demographics (age, sex), blood pressure medical history (heart disease, hypertension, stroke, and diabetes) were collected. Ischemic cardiomyopathy was confirmed if there was a history of coronary artery disease, angina or myocardial infarction. Hypertension was diagnosed if systolic blood pressure (SBP) ≥140 mmHg or diastolic blood pressure (DPB) ≥ 90 mmHg or history of antihypertensive therapy. Diabetes was defined if nonfasting plasma glucose ≥11.1 mmol/L or self-reported hypoglycemic therapy. Ischemic or hemorrhagic cerebral stroke history was also recorded. Blood biochemical biomarkers such as serum albumin, creatinine, C-reactive protein (CRP), mean corpuscular volume (MCV), red cell distribution width, alkaline phosphatase, blood glucose, white blood cell count, lymphocyte percentage and serum lipid fractions such as high-density lipoprotein cholesterol (HDL-c), low density lipoprotein cholesterol (LDL-c), total cholesterol (TC), triglyceride (TG) and aspartate aminotransferase (AST) were collected. Multiple lesions were evaluated on magnetic resonance imaging.

### 2.3 Stroke severity and disability assay

The severity of recent stroke was assessed using the NIHSS questionnaire scale by two experienced neurologists at admission. A consensus score through negotiations was made when there was a disagreement for the NIHSS evaluation. All the patients were classified into two groups based on NIHSS (NIHSS < 5 or NIHSS ≥ 5; NIHSS < 10 or NIHSS ≥ 10). The functional outcome was evaluated by the modified Rankin Scale (mRS) at admission and discharge. The mRS is a widely used measure of disability scored from 0 to 6, in which scored 0–1 indicates a good outcome with no disability, scored 2–5 indicates an unfavorable outcome with increasing amounts of disability, and scored 6 indicates death [28]. mRS ≥ 4 at discharge were considered as a severe disability.

### 2.4 Assessment of PhenoAge

PhenoAge was calculated according to the established method [19,20]. Briefly, chronological age and nine clinical biomarkers, including blood glucose, serum creatinine, serum albumin, C-reactive protein (CRP), lymphocyte percent, MCV, red cell distribution width, alkaline phosphatase, and white blood cell count were collected and the phenotypic age was calculated according to the following formula:

PhenoAge = 141.50225 + ln (−0.00553 × ln (exp (−1.51714 × exp (xb) 0.0076927))) 0.09165

Xb = −19.907 − 0.0336 × albumin + 0.0095 × creatinine + 0.1953 × glucose +0.0954 × ln (C-reactive protein) −0.012 × lymphocyte percentage +0.0268 × mean corpuscular volume + 0.3306 × erythrocyte distribution width +0.00188 × alkaline phosphatase + 0.0554 × leukocytes count + 0.0804 × chronological age. We also calculated the age gap: PhenoAge - chronological age. Age gap [29,30] and residual discrepancies between phenotypic age and chronological age [31] had been used to define accelerated aging. In this study we defined accelerated aging by both age gap at the third quartile and residual discrepancies between phenotypic age and chronological age (phenotypically older, positive value; phenotypically younger, negative value) [22,25,31].

### 2.5 Statistical analysis

All analyses were performed using SPSS (version 26.0) or R (version 3.6.3). The data were presented as means ± standard deviation (SD) for continuous variables and percentages for categorical variables. Normally distributed data were evaluated using independent-samples T-test, and abnormally distributed data were analyzed using Mann–Whitney U-test or Kruskal–Wallis test. Chi-squared test was used to compare the categorical variables. Multivariable logistic regression analyses were applied to estimate the relationship between Phenotype age, accelerated aging and the severity and disability of AIS. The performance of PhenoAge and chronological age in predicting stroke severity was evacuated by receiver operating characteristic (ROC) curve.

Firstly, we analyzed the difference of traditional demographics factors, previous medical history, blood biochemical biomarkers, serum lipid fractions, AST, SBP, DBP and PhenoAge between patients with low and high NIHSS. Covariates were chosen based on a priori knowledge of the potential associated factors of stroke severity. Those variables were used for calculating phenotypic age were not included in multivariable regression analysis. Three models were established to show the association between PhenoAge and the severity of AIS: model 1 included seven variables (PhenoAge, gender, multiple lesions, history of hypertension, diabetes, chronic kidney disease and heart diseases); model 2 further adjusted with chronological age; model 3 also included AST, HDL, LDL, TG and TC. Subgroup analyses were performed in patients with and without stroke history. Two-tailed P < 0.05 was considered statistically significant.

## 3 Results

### 3.1 Characteristic of patients

**Fig. 2:**
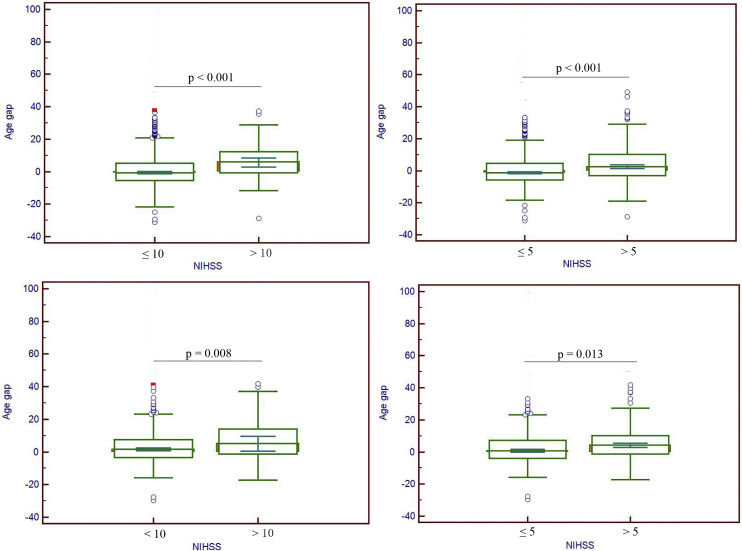
The age gap in patients with moderate-severe stroke (NIHSS ≥ 5 or NIHSS ≥ 10) and minor stroke (NIHSS < 5 or NIHSS < 10) in training (upper) and validation population (lower).

The characteristics of patients are shown in Table 1. 272 patients had a NIHSS ≥ 5 and 74 patients had a NIHSS ≥ 10. The age of patients with NIHSS ≥ 5 were significantly older than those with NIHSS < 5 (p < 0.001). Similarly, the age of patients with NIHSS ≥ 10 were significantly older than those with NIHSS < 10 (p < 0.001). Significant differences were also observed in sex, white blood cell count, MCV, serum albumin and CRP between those with high and low NIHSS (p < 0.05 or 0.001). Patients with NIHSS ≥ 5 also had higher level of alkaline posphatase (p = 0.04), blood glucose (p = 0.013) and lower level of lymphocyte percentage (p = 0.017) than those with NIHSS < 5. In addition, the PhenoAge in patients with high NIHSS (≥5 or ≥10) was significantly older than those with low NIHSS (<5 or <10). Moreover, the PhenoAge was higher than the chronological age in patients high NIHSS (≥5 or ≥10) (p < 0.001). However, no such differences were observed in patients with low NIHSS (<5 or <10). The number of phenotypically older patients in high NIHSS groups was greater than that in low NIHSS groups (p < 0.001). The age gap in patients high NIHSS (≥5 or ≥10) were significantly higher than those with low NIHSS (Fig. 2).

**Table 1:** Characteristic of patients based on NIHSS.

Variables	NIHSS ≥ 5 (n = 272)	NIHSS < 5 (n = 664)	P	NIHSS ≥ 10 (n = 74)	NIHSS < 10 (n = 862)	P
Age (years)	72.36 ± 12.68	67.41 ± 12.02	< 0.001	78.01 ± 12.00	68.06 ± 12.14	< 0.001
Sex (M)	158	476	< 0.001	36	598	< 0.001
Hypertension (yes)	203	505	0.78	51	657	0.68
Diabetes (yes)	124	264	0.08	29	359	0.94
Stroke history (yes)	92	212	0.51	25	279	0.49
Heart disease (yes)	77	152	0.06	30	199	< 0.001
CKD	6	10	0.45	1	15	1.0
Serum albumin (g/L)	38.47 ± 5.05	40.24 ± 5.23	< 0.001	37.09 ± 4.78	39.95 ± 5.21	< 0.001
Serum creatinine (μmol/L)	79.12 ± 38.83	80.06 ± 41.62	0.75	83.17 ± 52.94	79.49 ± 39.61	0.746
CRP (mg/L)	14.00 ± 29.06	7.39 ± 18.95	< 0.001	18.05 ± 26.24	8.56 ± 22.06	< 0.001
Mean corpuscular volume (fL)	91.35 ± 6.81	90.11 ± 7.10	0.014	92.75 ± 5.58	90.27 ± 7.11	0.004
Red cell distribution width (%)	13.18 ± 1.28	13.09 ± 1.90	0.50	13.41 ± 1.34	13.09 ± 1.77	0.13
Alkaline Posphatase (U/L)	87.04 ± 42.76	81.90 ± 30.75	0.04	89.89 ± 42.38	82.84 ± 33.94	0.09
Blood glucose (mmol/L)	7.04 ± 3.22	6.52 ± 2.81	0.013	7.29 ± 3.37	6.62 ± 2.90	0.06
White Blood Cell Count (×10^9^)	8.53 ± 5.61	7.15 ± 2.58	< 0.001	8.16 ± 3.44	7.50 ± 3.79	< 0.001
Lymphocyte percentage (%)	21.71 ± 25.56	24.52 ± 10.45	0.017	23.83 ± 46.33	23.69 ± 10.46	0.94
HDL (mmol/L)	1.28 ± 0.29	1.25 ± 0.28	0.28	1.32 ± 0.28	1.26 ± 0.28	0.13
LDL (mmol/L)	2.72 ± 0.88	2.69 ± 0.86	0.62	2.67 ± 0.91	2.70 ± 0.86	0.77
TC (mmol/L)	4.41 ± 1.09	4.34 ± 1.07	0.40	4.37 ± 1.11	4.36 ± 1.08	0.95
TG (mmol/L)	1.70 ± 2.13	1.59 ± 0.96	0.29	1.81 ± 3.91	1.61 ± 1.00	0.29
AST (U/L)	24.83 ± 24.89	21.94 ± 24.22	0.11	30.53 ± 30.66	22.17 ± 23.79	0.008
SBP (mmHg)	147.70 ± 24.21	147.13 ± 23.72	0.75	147.80 ± 28.76	147.26 ± 23.44	0.85
DPB (mmHg)	81.01 ± 13.84	82.61 ± 13.86	0.13	79.09 ± 16.76	82.42 ± 13.59	0.06
Phenotype age (years)	76.58 ± 16.83	67.99 ± 16.06	< 0.001	84.30 ± 14.63	69.30 ± 16.39	< 0.001
Residual discrepancies*	7.06 (-4.84−8.88)	−2.75 (−7.39−3.43)	< 0.001	4.06 (−2.72−10.49)	−2.45 (−7.26−3.67)	< 0.001
Phenotypically older Patients	110	259	< 0.001	48	321	< 0.001

AST: aspartate aminotransferase; CKD: chronic kidney disease; CRP: C-reactive protein; DBP: diastolic blood pressure; HDL-c: high-density lipoprotein cholesterol; LDL-c: low density lipoprotein cholesterol; NIHSS: National Institute of Health stroke scale; SBP: systolic blood pressure; TC: total cholesterol; TG: triglyceride.

* Between phenotypic age and chronological age and was shown as median (IQR).

The characteristics of patients in the validation cohort are shown in Supplemental Table S1. Similar to the trends observed in the training cohort, significant differences were found in age (p = 0.001), sex (p = 0.001), heart disease (p = 0.007), serum albumin (p = 0.004), CRP (p = 0.021), white blood cell count (p < 0.001), and lymphocyte percentage (p < 0.001) between patients with NISS ≥ 5 and NISS < 5. Additionally, significant differences in age (p = 0.034), sex (p = 0.001), heart disease (p < 0.001), serum albumin (p = 0.018), CRP (p = 0.01), MCV (p = 0.004), alkaline phosphatase (p = 0.039), and TG (p = 0.02) between patients with NISS ≥ 10 and NISS < 10 were observed. Similar differences in PhenoAge were also observed in the validation cohort.

### 3.2 The association between PhenoAge and stroke severity

Logistic regression analysis was applied to respectively demonstrate the relationship between phenotype age and stroke severity (Table 2). PhenoAge (odds ratio (OR) = 1.03, 95% confidence interval (CI): 1.02–1.04, for NIHSS ≥ 5; OR = 1.05, 95%CI: 1.03–1.07, for NIHSS ≥ 10) and multiple lesions (OR = 1.67, 95%CI: 1.1–2.34, for NIHSS ≥ 5; OR = 2.98, 95%CI: 1.45−6.12, for NIHSS ≥ 10) were independently associated with high NIHSS in all three models.

**Table 2:** Association between phenotype age and stroke severity.

		Model 1	Model 2	Model 3
NIHSS ≥ 5	Phenotype age	1.03 (1.02−1.04)	1.03 (1.01−1.04)	1.02 (1.01−1.04)
	Sex (male vs. female)	0.63 (0.44−0.90)	0.64 (0.45−0.92)	0.71 (0.48−1.04)
	Stroke history	1.05 (0.73−1.50)	1.05 (0.73−1.50)	1.09 (0.75−1.56)
	Hypertension	0.89 (0.58−1.35)	0.88 (0.58−1.34)	0.99 (0.64−1.53)
	Diabetes	1.03 (0.73−1.46)	1.05 (0.74−1.49)	0.95 (0.65−1.37)
	Heart disease	0.84 (0.56−1.27)	0.83 (0.55−1.26)	0.86 (0.56−1.33)
	CKD	0.94 (0.24−3.62)	0.96 (0.25−3.73)	1.09 (0.28−4.27)
	Multiple lesions	1.67 (1.19−2.34)	1.68 (1.20−2.36)	1.65 (1.16−2.35)
NIHSS ≥ 10	Phenotype age	1.05 (1.03−1.07)	1.05 (1.02−1.07)	1.04 (1.01−1.07)
	Sex (male vs. female)	0.49 (0.25−0.98)	0.53 (0.26−1.06)	0.55 (0.25−1.19)
	Stroke history	1.03 (0.51−2.10)	1.03 (0.51−2.08)	1.04 (0.48−2.23)
	Hypertension	0.72 (0.32−1.61)	0.71 (0.32−1.61)	0.91 (0.36−2.30)
	Diabetes	0.86 (0.43−1.73)	0.90 (0.45−1.82)	0.74 (0.34−1.63)
	Heart disease	1.17 (0.56−2.44)	1.10 (0.52−2.33)	1.27 (0.57−2.83)
	Multiple lesions	2.98 (1.45−6.12)	3.02 (1.48−6.19)	3.57 (1.60−7.98)

Model 2 was adjusted with chronological age. Model 3 was further adjusted with aspartate aminotransferase, high-density lipoprotein cholesterol, low density lipoprotein cholesterol, total cholesterol, triglyceride.

CKD: chronic kidney disease; NIHSS: National Institute of Health stroke scale.

We also showed the association between age gap (PhenoAge acceleration) and stroke severity (Table 3 and Supplemental Fig. S1). Patients who were in quartile 4 in terms of aging acceleration had the higher NIHSS compared with patients who were in quartile 1 (Supplemental Fig. S1). High age gap was associated with high probability of severe stroke. Each 1-year increase in PhenoAge acceleration increased the probability of NIHSS ≥ 5 by 3% and NIHSS ≥ 10 by 5% (Table 3). Patients who were in quartile 4 in terms of aging acceleration had the highest probability of NIHSS ≥ 5 or NIHSS ≥ 10 compared with patients who were in quartile 1 (OR = 2.60, 95%CI: 1.51−4.47; OR = 7.88, 95%CI: 2.04−30.56). The probability of NIHSS ≥ 5 or NIHSS ≥ 10 was significantly increased in individuals with accelerated aging (age gap > 6.4) versus individuals with no accelerated aging (OR = 1.79, 95%CI: 1.18−2.72; OR = 3.53, 95%CI: 1.60−7.77). Similar trends was observed when accelerated aging was defined by residual discrepancies between PhenoAge and chronological age (OR = 1.02, 95%CI: 1.01−1.04; OR = 1.05, 95%CI: 1.02−1.08). In addition, those patients that were phenotypically older also had higher probability of NIHSS ≥ 5 or NIHSS ≥ 10 than those that were phenotypically younger (OR = 2.01, 95%CI: 1.21−3.35; OR = 3.69, 95%CI: 1.36−10.0) (Table 3).

**Table 3:** Association between age gap and stroke severity.

		Model 1	Model 2	Model 2
NIHSS ≥ 5	Continuous	1.03 (1.01−1.04)	1.03 (1.01−1.04)	1.03 (1.008−1.04)
	Q1	1	1	1
	Q2	1.45 (0.87−2.41)	1.45 (0.87−2.41)	1.31(0.78−2.20)
	Q3	2.24 (1.35−3.72)	2.23 (1.34−3.71)	2.05(1.22−3.45)
	Q4	2.91 (1.73−4.88)	2.89 (1.72−4.86)	2.60 (1.51−4.47)
	Non-Accelerated ageing	1	1	1
	Accelerated ageing	1.86 (1.26−2.73)	1.85 (1.25−2.72)	1.79 (1.18−2.72)
	Phenotypically younger	1	1	1
	Phenotypically older	2.12 (1.32−3.39)	1.97 (1.38−2.82)	2.01 (1.21−3.35)
	Residual discrepancies	1.02 (1.01−1.04)	1.03 (1.01−1.04)	1.02 (1.01−1.04)
NIHSS ≥ 10	Continuous	1.04 (1.02−1.06)	1.04 (1.02−1.06)	1.05 (1.02−1.07)
	Q1	1	1	1
	Q2	3.01 (0.79−12.01)	3.06 (0.78−11.98)	2.03 (0.49−8.38)
	Q3	4.16 (1.07−16.23)	4.15 (1.06−16.21)	3.64(0.91−14.49)
	Q4	10.86 (2.96−39.82)	10.82 (2.94−39.78)	7.88 (2.04−30.56)
	Non-Accelerated ageing	1	1	1
	Accelerated ageing	3.98 (1.98−8.03)	3.96 (1.96−8.01)	3.53 (1.60−7.77)
	Phenotypically younger	1	1	1
	Phenotypically older	3.96 (1.62−9.72)	4.13 (1.98−8.65)	3.69 (1.36−10.0)
	Residual discrepancies	1.04 (1.02−1.06)	1.05 (1.02−1.07)	1.05 (1.02−1.08)

Model 1 was adjusted with sex, hypertension, stroke history, diabetes, chronic kidney disease, heart disease, lesions number.

Model 2 was further adjusted with chronological age.

Model 3 was further adjusted with aspartate aminotransferase, high-density lipoprotein cholesterol, low density lipoprotein cholesterol, total cholesterol, triglyceride.

Q1: < −5.26; Q2: −5.26−0; Q3:0−6.4; Q4: > 6.4.

Accelerated ageing means age gap >6.4.

NIHSS: National Institute of Health stroke scale.

Accelerated aging, Phenotypically older and residual discrepancies were separably included in models.

Then, we performed subgroup analysis on the patients with or without history of stroke (Table 4, Supplemental Table S2). The results showed that in patients with a previous stroke history, PhenoAge (OR = 1.03, 95%CI: 1.02–1.04 for NIHSS ≥ 5; OR = 1.08, 95%CI: 1.01–1.14 for NIHSS ≥ 10) and multiple lesions (OR = 2.83, 95%CI: 1.50−5.36 for NIHSS ≥ 10; OR = 13.21, 95%CI:1.41–123.74) were associated with a high NIHSS. For those patients without stroke history, PhenoAge (OR = 1.02, 95%CI: 1.00–1.04 for NIHSS ≥ 5; OR = 1.03, 95%CI: 1.00–1.08 for NIHSS ≥ 10) was also associated with a high NIHSS (Supplemental Table S2).

**Table 4:** Association between phenotype age and stroke severity (NIHSS ≥ 5) in patients with or without stroke history.

		Model 1	Model 2	Model 3
With stroke history	Phenotype age	1.02 (1.01−1.04)	1.04 (1.01−1.07)	1.04 (1.01−1.08)
	Sex (male vs. female)	0.52 (0.32−0.85)	1.05 (0.54−2.00)	1.46 (0.70−3.03)
	Hypertension	1.26 (0.72−2.22)	0.47 (0.23−1.06)	0.55 (0.26−1.66)
	Diabetes	0.84 (0.52−1.37)	0.96 (0.52−1.78)	0.84 (0.43−1.62)
	Heart disease	0.59 (0.34−1.02)	0.96 (0.49−1.88)	1.17 (0.57−2.38)
	CKD	2.94 (0.32−27.15)	0.71 (0.09−5.68)	0.57 (0.07−4.95)
	Multiple lesions	2.97 (1.64−5.37)	2.85 (1.56−5.18)	2.83 (1.50−5.36)
Without stroke history	Phenotype age	1.03 (1.02−1.04)	1.02 (1.01−1.04)	1.02 (1.00−1.04)
	Sex (male vs. female)	0.49(0.32−0.75)	0.52 (0.33−0.80)	0.53 (0.33−0.84)
	Hypertension	1.21 (0.71−2.06)	1.20 (0.70−2.04)	1.31 (0.74−2.30)
	Diabetes	1.03 (0.67−1.59)	1.08 (0.69−1.67)	0.97 (0.62−1.53)
	Heart disease	0.79 (0.46−1.36)	0.76 (0.44−1.32)	0.72 (0.41−1.27)
	CKD	0.67 (0.01−4.69)	0.71 (0.10−4.89)	0.84 (0.12−6.14)
	Multiple lesions	1.25 (0.82−1.91)	1.26 (0.83−1.92)	1.25 (0.81−1.93)

Model 2 was adjusted with chronological age. Model 3 was further adjusted with aspartate aminotransferase, high-density lipoprotein cholesterol, low density lipoprotein cholesterol, total cholesterol, triglyceride.

CKD: chronic kidney disease; NIHSS: National Institute of Health stroke scale.

### 3.3 The performance of PhenoAge in predicting stroke severity

The performance of PhenoAge in predicting stroke severity was analyzed using the ROC curves (Supplemental Fig. S2). The area under the ROC curve (AUC) of phenotype age was higher than that of chronological age in identifying patients with NIHSS ≥ 5 (0.66, 95%CI:0.62−0.70 vs. 0.61, 95%CI: 0.58−0.65, p < 0.01) (Supplemental Fig. S2A). Similar result was observed in identifying patients with NIHSS ≥ 10 (AUC, 0.69, 95%CI:0.60−0.77 vs. 0.63, 95%CI: 0.55−0.72, p = 0.05) (Supplemental Fig. S2B).

### 3.4 The association between accelerated aging and severe disability

Fifty-three patients had severe disabilities at discharge. The association between age gap/accelerated aging and severe disability at discharge is shown in Table 5. The probability of worse prognosis (mRS ≥ 4) was significantly increased in individuals with accelerated aging (age gap > 6.4) versus individuals with no accelerated aging (OR = 2.87, 95%CI: 1.09−7.53) after adjusting baseline NIHSS, mRS, chronological age and treatment. In addition, those patients that were phenotypically older had higher probability of worse prognosis than those that were phenotypically younger (OR = 4.88, 95%CI: 1.20−19.88). Similar trends was observed when accelerated aging was defined by residual discrepancies between PhenoAge and chronological age (OR = 1.06, 95%CI: 1.02−1.11). Sensitivity analysis in patients with routine therapy also showed similar results (OR = 3.57, 95%CI: 1.24−10.23; OR = 4.55, 95%CI: 1.03−20.05; OR = 1.06, 95%CI: 1.02−1.12).

**Table 5:** Association between accelerated aging and severe disability at discharge.

		Model 1	Model 2
All	Baseline NIHSS	1.13 (1.05−2.10)	1.13 (1.05−1.22)
	Baseline mRS	3.86 (2.16−6.92)	3.88 (2.16−6.94)
	Non-Accelerated aging	1	1
	Accelerated aging	2.87 (1.09−7.53)	2.87 (1.09−7.53)
	Phenotypically younger	1	1
	Phenotypically older	4.70 (1.15−19.26)	4.88 (1.20−19.88)
	Residual discrepancies	1.07 (1.03−1.11)	1.06 (1.02−1.11)
Routine therapy (n = 792)	Baseline NIHSS	1.12 (1.03−1.21)	1.12 (1.03−1.21)
	Baseline mRS	4.96 (2.45−10.05)	4.98 (2.45−10.11)
	Non-Accelerated aging	1	1
	Accelerated aging	3.56 (1.24−10.24)	3.57 (1.24−10.23)
	Phenotypically younger	1	1
	Phenotypically older	4.34 (1.00−18.92)	4.55 (1.03−20.05)
	Residual discrepancies	1.07 (1.02−1.12)	1.06 (1.02−1.12)

Model 1 was adjusted with sex, hypertension, stroke history, diabetes, chronic kidney disease, liver function, heart disease, lesions number.

Model 2 was further adjusted with chronological age.

Accelerated ageing means age gap >6.4.

mRS: modified Rankin Scale; NIHSS: National Institute of Health stroke scale.

Accelerated aging, Phenotypically older and residual discrepancies were separably included in models.

### 3.5 Validation cohort

In the validation cohort with 512 patients, the multivariable logistic regression analysis revealed that PhenoAge was associated with NIHSS ≥ 5 (OR = 1.02, 95%CI: 1.003–1.04) (Supplemental Table S3) and NIHSS ≥ 10 (OR = 1.04, 95%CI: 1.006–1.07) (Supplemental Table S3). ROC curve also showed that PhenoAge had higher performance in identifying patients with high NIHSS (AUC, 0.65, 95%CI:0.60−0.70 vs. 0.61, 95%CI: 0.56−0.67, p = 0.009; AUC, 0.70, 95%CI:0.60−0.80 vs. 0.65, 95%CI: 0.55−0.71, p = 0.06) (Supplemental Fig. S2C, 2D). The probability of NIHSS ≥ 5 or NIHSS ≥ 10 was significantly increased in individuals with accelerated aging (age gap > 6.4) versus individuals with no accelerated aging (OR = 1.51, 95%CI: 0.98–2.71; OR = 2.68, 95%CI: 1.03–6.98). Similar trends was observed when accelerated aging was defined by residual discrepancies between PhenoAge and chronological age (OR = 1.04, 95%CI: 1.01–1.06). In addition, those patients that were phenotypically older also had higher probability of NIHSS ≥ 5 or NIHSS ≥ 10 than those that were phenotypically younger (OR = 2.03, 95%CI: 1.33–3.11; OR = 2.66, 95%CI: 1.32–5.33).

The probability of severe disability (mRS ≥ 4) was significantly increased in individuals with accelerated aging (age gap >6.4) versus individuals with no accelerated aging (OR = 5.01, 95%CI: 1.92–13.11) after adjusting baseline NIHSS, mRS, chronological age and treatment. Similar trends was observed when accelerated aging was defined by residual discrepancies between PhenoAge and chronological age (OR = 1.06, 95%CI: 1.02–1.10). In addition, those patients that were phenotypically older had higher probability of worse prognosis than those that were phenotypically younger (OR = 1.79, 95%CI: 0.99–3.46).

## 4 Discussion

Stroke severity is associated with the prognosis and clinical outcome in patients with AIS. Biological age may be more accurate than chronological age in determining chronic health outcomes. In this large, retrospective cohort study, we showed that PhenoAge in patients with high NIHSS (≥5 or ≥10) were significantly higher than the chronological age. PhenoAge and accelerated aging were significantly associated with stroke severity and the probability of severe disability even after adjusting for chronological age. Moreover, PhenoAge exhibited better performance in predicting a more severe stroke than chronological age alone. Additionally, we validated our results in an independent population and consistently found that PhenoAge was associated with stroke severity and the probability of severe disability at discharge.

Chronological age has been considered as the strongest risk factor for aging-related death and disease [20,32]. However, individuals with the same chronological age may have different susceptibilities to age-related diseases and outcome [32], which indicated that they may have different biological age. Many studies have focused on the modified effect of biological age on disease occurrence, prognosis and outcomes [19,[22], [23], [24], [25]]. Interestingly, several studies also showed that biological age was associated with stroke recurrence, 3-month outcome in ischemic stroke and stroke mortality [26,27,33,34]. However, biological age used in those studies is genetic aging markers derived from DNA methylation (DNAm) techniques. DNA methylation detection is not a routine clinical test [24]. Recent studies have demonstrated that a novel aging marker “PhenoAge” which is composed of chronological age and several serum biomarkers [19,20,[22], [23], [24]]. These studies also showed that PhenoAge is associated with health outcome, such as mortality, disease severity and occurrences. To our knowledge, the present study is the first to evaluate the role of blood chemistry-based aging markers in AIS patients. Our results demonstrated that PhenoAge was associated with AIS severity and PhenoAge-based age was superior to chronological age in predicting AIS severity. Our study offers a promising indicator for the administration and management of AIS.

Some studies have also investigated the association between accelerated aging and health risks or mortality. PhenoAge acceleration was associated with common chronic respiratory diseases and the risk of incident rheumatoid arthritis [25,35]. Accelerated aging may partially mediate the associations of lifestyles with cardiovascular disease, cancer, and mortality [36]. Our results indicate that the probability of severe stroke is significantly higher in patients with accelerated aging compared to those without accelerated aging. Stroke is a leading cause of adult disability worldwide [37]. Age is an independent risk factor of disability after minor stroke [38]. Our data also demonstrate that accelerated aging is significantly associated with severe disability at discharge. The mechanisms by which accelerated aging affects health are complex and have not yet been fully elucidated. Genetic differences may contribute to differential vulnerability to aging and disease [39]. PhenoAge acceleration-related single nucleotide polymorphisms play an important role in biological processes involved in immune system process and chronic inflammatory [40]. Chronic inflammation is closely associated with stroke.

Associated factors with stroke severity have also been investigated in many studies. High white matter hyperintensity burden [15], neutrophil counts and neutrophil-to-lymphocyte ratio [16], systemic immune-inflammation index and system inflammation response index [41], and stroke subtype (embolic stroke and thrombotic stroke) [18] have been shown to be associated with stroke severity. Several studies also showed the role of serum albumin in stroke [42,43] and cardiovascular complications [44]. Our data also showed significant differences between serum albumin and inflammatory markers between patients with high and low NIHSS. It has been shown that as the body ages biologically, levels of tumour necrosis factor, interleukin 6, C-reactive protein, other inflammatory molecules will increased [35]. PhenoAge is also calculated based on several inflammatory markers including CRP, lymphocyte percent and white blood cell count, and serum albumin, which may partially explain why PhenoAge is associated with stroke severity. However, further studies should be performed to clarify the causal relationship between PhenoAge and stroke severity.

Our study has several advantages. First, the sample size is large. There were 936 patients in cohort one and 512 in cohort two. Second, we validated the results in an independent population. Our conclusion will be more stronger than that came from only one population. However, our study also has several limitations to be acknowledged. First, we did not analyze the association between PhenoAge and a higher NIHSS, such as NIHSS > 15 or NIHSS > 21, which indicated a more severe condition [45], because the sample size of those patients was relatively small (n < 30). Second, we did not show the association between PhenoAge and short or long stroke outcomes because the follow-up data was not obtained. Third, although we controlled for several variables, residual confounding effects from other factors, such as medications, smoking and drinking habits, as well as body mass index, cannot be ruled out. Fourth, this is a single center study. Although we had performed an internal validation, our results should be validated in an external population. Fifth, PhenoAge may be modified by the acute stroke episode if stroke episode can influence any of the markers for PhenoAge calculation. However, it is difficult to determine whether stroke episode causes changes in blood biochemistry or blood biochemistry causes changes in stroke episode. Prospective or longitudinal studies may be needed to show the causal relationship between phenotypic age and accelerated aging with stroke severity. Finally, the performance of phenotypic age alone for stroke severity or disability prediction was relatively low. Phenotype age alone may not be sufficient for estimating the severity of stroke.

In conclusion, our data demonstrate that PhenoAge and accelerated aging, calculated from routinely available laboratory measurements, are associated with stroke severity and severe disability at discharge in patients with AIS. Additionally, PhenoAge outperforms chronological age in predicting AIS severity. Thus, PhenoAge or accelerated aging could serve as a useful tool for identifying the more severe population and managing AIS.

## CRediT authorship contribution statement

YL, JW, ZW: collection of data, interpretation and analysis of the data, and review of the manuscript. YW, MW, RC: interpretation and analysis of the data, and review of the manuscript. JW, XC: study design, interpretation and analysis of the data, and review of the manuscript. All authors approved the final manuscript.

## Consent for publication

Not applicable.

## Ethics approval and consent to participate

Declaration of Helsinki was followed during the study. This study was approved by our Institutional Ethics Committee and no written informed consent was required.

## Declaration of Generative AI and AI-assisted technologies in the writing process

Not applicable.

## Funding

None.

## Availability of data and materials

The datasets used and/or analyzed during the current study available from the corresponding author on reasonable request.

## Declaration of competing interest

The authors declare that they have no competing interests.
