# Supplementary material for: Association of phenotypic age and accelerated aging with severity and disability in patients with acute ischemic stroke
Source: J Nutr Health Aging. 2024 Nov 2;28(12):100405. doi: 10.1016/j.jnha.2024.100405 (PMC12877281; doi:10.1016/j.jnha.2024.100405)

# **Association of Phenotypic Age and Accelerated Aging with Severity and Disability in Patients with Acute Ischemic Strok****e**

Supplemental Table 1 Characteristic of patients based on NISS in validation population

| Variables | NIHSS ≥ 5 (n = 153) | NIHSS < 5  (n = 359) | P | NIHSS ≥ 10  (n = 47) | NIHSS < 10  (n = 465) | P |
| --- | --- | --- | --- | --- | --- | --- |
| Age (years) | 71.78 ± 13.18 | 67.41 ± 12.02 | 0.001 | 72.84 ± 13.66 | 68.08 ± 13.19 | 0.034 |
| Sex (M) | 72 | 208 | 0.001 | 19 | 261 | 0.001 |
| Hypertension (yes) | 89 | 207 | 0.88 | 29 | 267 | 0.69 |
| Diabetes (yes) | 48 | 93 | 0.22 | 11 | 130 | 0.39 |
| Stroke history (yes) | 44 | 102 | 0.97 | 12 | 134 | 0.51 |
| Heart disease (yes) | 35 | 49 | 0.007 | 16 | 68 | < 0.001 |
| Serum albumin (g/L) | 37.34 ± 5.00 | 39.04 ± 5.47 | 0.004 | 37.09 ± 4.78 | 39.95 ± 5.21 | 0.018 |
| Serum creatinine (μmol/L) | 77.11 ± 43.85 | 77.86 ± 30.30 | 0.75 | 83.17 ±52.94 | 79.49 ± 39.61 | 0.64 |
| CRP (mg/L) | 13.67 ± 23.47 | 8.22 ± 20.39 | 0.021 | 18.12 ± 28.27 | 8.97 ± 20.44 | 0.01 |
| Mean corpuscular volume (fL) | 90.35 ± 7.83 | 89.16 ± 8.75 | 0.20 | 88.98 ± 10.84 | 89.47 ± 8.20 | 0.004 |
| Red cell distribution width (%) | 13.11 ± 1.09 | 13.22 ± 2.51 | 0.65 | 13.35 ± 1.39 | 13.17 ± 2.25 | 0.62 |
| Alkaline Posphatase (U/L) | 82.73 ± 43.21 | 76.32 ± 27.24 | 0.07 | 88.61 ± 65.07 | 77.13 ± 27.19 | 0.039 |
| Blood glucose (mmol/L) | 6.93 ± 3.23 | 6.67 ± 2.89 | 0.43 | 6.74 ± 3.35 | 6.75 ± 2.96 | 0.99 |
| White Blood Cell Count (×10^9^) | 8.10 ± 3.00 | 6.98 ± 2.34 | < 0.001 | 7.97 ± 3.31 | 7.25 ± 2.51 | 0.11 |
| Lymphocyte percentage (%) | 19.44 ± 10.28 | 24.67 ± 10.32 | < 0.001 | 19.93 ± 13.78 | 23.42 ± 10.12 | 0.94 |
| HDL (mmol/L) | 1.26 ± 0.30 | 1.25 ± 0.29 | 0.92 | 1.31 ± 0.35 | 1.25 ± 0.29 | 0.30 |
| LDL (mmol/L) | 2.80 ± 0.98 | 2.83 ± 0.89 | 0.82 | 2.68 ± 1.09 | 2.83 ± 0.89 | 0.39 |
| TC (mmol/L) | 4.34 ± 1.10 | 4.33 ± 1.06 | 0.90 | 4.30 ± 1.34 | 4.34 ± 1.04 | 0.85 |
| TG (mmol/L) | 1.89 ± 3.07 | 1.64 ± 0.98 | 0.25 | 2.44 ± 5.39 | 1.64 ± 1.03 | 0.02 |
| AST (U/L) | 27.67 ± 28.04 | 24.85 ± 27.68 | 0.37 | 33.85 ± 36.99 | 24.86 ± 26.57 | 0.07 |
| SBP (mmHg) | 148.03 ± 24.29 | 146.14 ± 25.34 | 0.50 | 146.58 ± 28.60 | 146.73 ± 24.66 | 0.97 |
| DPB (mmHg) | 79.31 ± 13.89 | 79.94 ± 14.27 | 0.69 | 79.61 ± 16.67 | 79.77 ±13.87 | 0.95 |
| Phenotype age | 76.69 ± 16.94 | 69.42 ± 17.18 | < 0.001 | 81.28 ± 17.76 | 71.49 ± 16.53 | < 0.001 |
| Residual discrepancies* | 3.90 (-5.07-6.44) | -2.86 (-7.64-3.44) | < 0.001 | 1.44 (-5.06-10.41) | -2.18 (-7.19-3.73) | 0.013 |
| Phenotypically older Patients | 79 | 109 | <0.001 | 26 | 179 | 0.025 |

AST: aspertate aminotransferase; CRP: C-reactive protein; DBP: diastolic blood pressure; HDL-c: high-density lipoprotein cholesterol; LDL-c: low density lipoprotein cholesterol; SBP: systolic blood pressure; TC: total cholesterol; TG: triglyceride;

* between phenotypic age and chronological age and was shown as median (IQR)

Supplemental Table 2 Association between phenotypic age and stroke severity (NIHSS ≥ 10 ) in patients without stroke history

|  |  | Model 1 | Model 2 | Model 2 |
| --- | --- | --- | --- | --- |
| without stroke history | Phenotype age | 1.06 (1.03-1.08) | 1.04 (1.002-1.08) | 1.03 (1.00-1.08) |
|  | Sex (male vs female) | 0.44 (0.18-1.07) | 0.57 (0.23-1.40) | 0.42 (0.15-1.16) |
|  | Hypertension | 0.88 (0.30-2.56) | 0.85 (0.29-2.52) | 1.18 (0.31-4.43) |
|  | Diabetes | 1.05 (0.43-2.54) | 1.33 (0.54-3.32) | 1.14 (0.42-3.09) |
|  | Heart disease | 1.63 (0.64-4.08) | 1.23 (0.47-3.19) | 1.17 (0.41-3.31) |
|  | CKD | / | / | / |
|  | Multiple lesions | 2.38 (0.98-5.79) | 2.43 (0.99-5.93) | 2.28 (0.87-6.00) |
| with stroke history | Phenotype age | 1.04 (1.00-1.08) | 1.08 (1.03-1.13) | 1.08 (1.01-1.14) |
|  | Sex (male vs female) | 0.60 (0.20-1.83) | 0.68 (0.21-2.22) | 0.94 (0.23-3.82) |
|  | Hypertension | 0.59 (0.17-2.01) | 0.46 (0.13-1.71) | 0.68 (0.15-3.15) |
|  | Diabetes | 0.68 (0.22-2.12) | 0.45 (0.13-1.60) | 0.36 (0.08-1.69) |
|  | Heart disease | 0.75 (0.22-2.12) | 1.13 (0.31-4.13) | 1.73 (0.40-7.42) |
|  | CKD | / | / | / |
|  | Multiple lesions | 5.02 (1.35-18.69) | 4.28(1.12-16.36) | 13.21 (1.41-123.74) |

Model 2 was adjusted with chronological age. Model 3 was adjusted with aspartate aminotransferase, high-density lipoprotein cholesterol, low density lipoprotein cholesterol, total cholesterol, triglyceride

.

Supplemental Table 3 Association between phenotype age and stroke severity in validation population

|  |  | Model 1 | Model 2 | Model 3 |
| --- | --- | --- | --- | --- |
| NIHSS ≥ 5 | Phenotype age | 1.02 (1.003-1.04) | 1.03 (1.01-1.05) | 1.03 (1.003-1.05) |
|  | Sex (male vs female) | 0.56 (0.32-0.97) | 0.59 (0.36-0.96) | 0.70 (0.41-1.19) |
|  | Stroke history | 1.02 (0.59-1.75) | 0.89 (0.55-1.43) | 1.06 (0.60-1.88) |
|  | Hypertension | 0.78 (0.42-1.45) | 0.89 (0.51-1.53) | 1.09 (0.62-1.95) |
|  | Diabetes | 1.02 (0.58-1.78) | 1.23 (0.86-1.96) | 0.99 (0.60-1.62) |
|  | Heart disease | 1.13 (0.58-2.20) | 1.20 (0.76-1.91) | 1.18 (0.59-2.35) |
|  | CKD | 0.43 (0.05-3.95) | 0.36 (0.04-3.41) | 0.49 (0.058-4.61) |
|  | Multiple lesions | 1.02 (0.61-1.73) | 1.48 (0.94-2.32) | 1.10 (0.64-1.90) |
| NIHSS ≥ 10 | Phenotype age | 1.03 (1.001-1.05) | 1.04 (1.01-1.07) | 1.04 (1.006-1.07) |
|  | Sex (male vs female) | 0.46 (0.20-1.05) | 0.41 (0.17-1.68) | 0.49 (0.18-1.29) |
|  | Stroke history | 0.63 (0.26-1.53) | 0.68 (0.27-1.68) | 0.62 (0.23-1.72) |
|  | Hypertension | 0.80 (0.32-2.01) | 0.82 (0.33-2.08) | 1.27 (0.41-3.88) |
|  | Diabetes | 0.56 (0.23-1.36) | 0.59 (0.24-1.45) | 0.44(0.16-1.25) |
|  | Heart disease | 2.02 (0.84-4.89) | 2.08 (0.84-5.13) | 2.41 (0.90-6.43) |
|  | Multiple lesions | 1.82 (0.81-4.11) | 1.86 (0.82-4.25) | 1.75 (0.70-4.35) |

Model 2 was adjusted with chronological age. Model 3 was adjusted with aspartate aminotransferase, high-density lipoprotein cholesterol, low density lipoprotein cholesterol, total cholesterol, triglyceride

Supplemental Figure 1. NIHSS in patients with different age gaps. Q1: < -5.26; Q2:-5.26-0; Q3:0-6.4; Q4: > 6.4


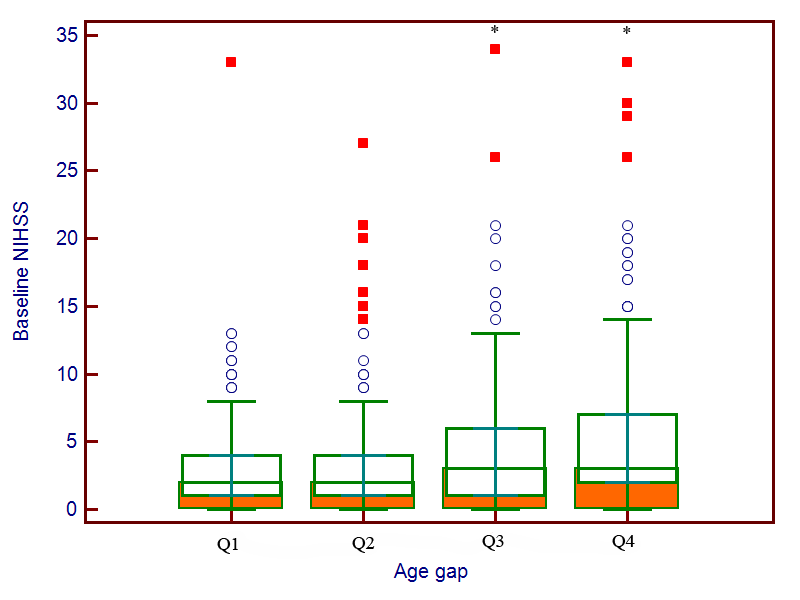


Supplemental Figure 2. Receiver operating characteristic curve to show the performance of phenotype age and chronological age in predicting stroke severity. A: NIHSS ≥ ; B: NIHSS ≥ 10; C: NIHSS ≥ 5 in validation group; D: NIHSS ≥ 10 in validation group


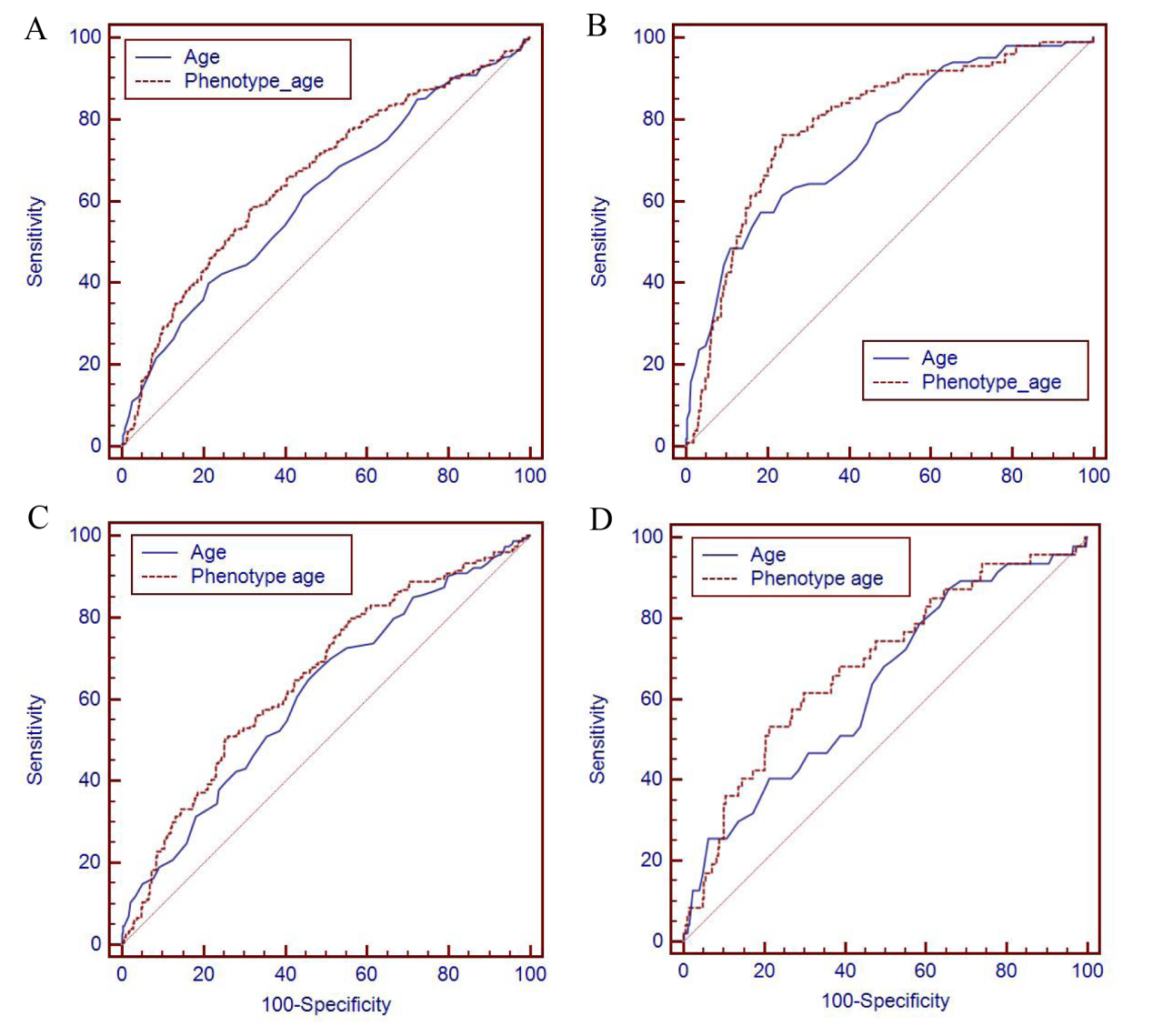

Supplement: Supplementary file 1 [file mmc1.docx]
